# Cost-effectiveness of scar management post-burn: a trial-based economic evaluation of three intervention models

**DOI:** 10.1038/s41598-022-22488-3

**Published:** 2022-11-03

**Authors:** Steven M. McPhail, Jodie Wiseman, Megan Simons, Roy Kimble, Zephanie Tyack

**Affiliations:** 1 Australian Centre for Health Services Innovation and Centre for Healthcare Transformation, School of Social Work and Public Health, Queensland University of Technology, 149 Victoria Park Rd, Kelvin Grove, QLD 4059 Australia; 2 Digital Health and Informatics Directorate, Metro South Health, 199 Ipswich Road, Brisbane, Australia; 3 Centre for Children’s Burns and Trauma Research, Child Health Research Centre, The University of Queensland, 62 Graham St, South Brisbane, QLD Australia; 4 Department of Occupational Therapy, Queensland Children’s Hospital, 501 Stanley St, South Brisbane, QLD Australia; 5 Pegg Leditschke Children’s Burns Centre, Queensland Children’s Hospital, 501 Stanley St, South Brisbane, QLD Australia

**Keywords:** Trauma, Health care economics, Paediatrics

## Abstract

Optimal burn scar management has the potential to markedly improve the lives of children, but can require substantial healthcare resources. The study aimed to examine the cost-effectiveness of three scar management interventions: pressure garment; topical silicone gel; combined pressure garment and topical silicone gel therapy, alongside a randomised controlled trial of these interventions. Participants were children (n = 153) referred for burn scar management following grafting, spontaneous healing after acute burn injury, or reconstructive surgery. Healthcare resource use was costed from a health service perspective (6-months post-burn time-horizon). The mean total scar management cost was lowest in the topical silicone gel group ($382.87 (95% CI $337.72, $443.29)) compared to the pressure garment ($1327.02 (95% CI $1081.46, $1659.95)) and combined intervention $1605.97 ($1077.65, $2694.23)) groups. There were no significant between-group differences in Quality Adjusted Life Year estimates. There was a 70% probability that topical silicone gel dominated pressure garment therapy (was cheaper and more effective), a 29% probability that pressure garment therapy dominated combined therapy, and a 63% probability that topical silicone gel dominated combined therapy. In conclusion, topical silicone gel was the cheaper intervention, and may be favoured in the absence of clear clinical effect favouring pressure garment therapy or a combination of these management approaches.

Trial registration: ACTRN12616001100482 (prospectively registered).

## Introduction

Managing scarring after a burn injury in children remains a substantial challenge for multidisciplinary rehabilitation teams worldwide despite a global reduction in burn injury incidence, mortality, and severity of burns over the last two decades^1^. Of children attending a specialist hospital with a burn, recent estimates indicate approximately one third will develop hypertrophic scars^2,3^. The burden of burn scars arises not only from the visible differences in appearance in children, which can be stigmatising and reduce school attendance, but also from the burden of conservative first line scar interventions on the daily lives of families.

Pressure garments, topical silicone gels, splints, exercise and moisturisers are commonly used first line interventions for scar management^4^, which may take families several hours per day to implement. Scar interventions often commence around the time of wound healing to prevent or treat scars and may continue for months after the burn injury if the scar does not mature earlier. The effectiveness of many of these interventions is unclear^5^ and few trials have been conducted in children^6^.

A recent three group randomised controlled trial (RCT) among children referred for post-burn scar management investigated the comparative effect of three scar management approaches (pressure garment; topical silicone gel; combined pressure garment and topical silicone gel)^7^. Notably, no additional benefit was identified from combining topical silicone and pressure interventions on scar thickness or itch, compared to the topical silicone gel or pressure interventions alone, nor between the individual interventions (pressure alone versus topical silicone gel alone). In the absence of high-quality evidence of superior clinical effectiveness, healthcare resource use and costs associated with interventions are likely to be an important consideration for healthcare services seeking to provide high value healthcare^8^. Scar management for children recovering from burns is an important case-in-point where healthcare resource use and costs may differ substantially between alternative intervention approaches for which there is no clinical trial evidence favouring a particular intervention approach.

The healthcare cost of burn rehabilitation has not typically been the focus of previous economic evaluations for burn injuries and burn rehabilitation interventions^9^. This is despite burn rehabilitation expenses being identified as potentially more than four times higher than costs for acute burn management^10^. The most studied costs related to burn care identified in a 2014 systematic review were acute hospitalisations, followed by dressings, medications, and surgery^9^. Further, economic evaluations focused specifically on paediatric burns have largely focused on wound dressings^11,12^ or the healthcare costs of managing a burn up to 6-weeks post-injury^13^, not on scar management. Systematic review searches conducted by the authors did not identify any economic evaluation of the cost-effectiveness of pressure garment and topical silicone gel therapy^4^.

This paper aimed to examine the incremental cost-effectiveness (cost-utility analysis) of three scar management intervention approaches: (1) pressure garment; (2) topical silicone gel; (3) combined pressure garment and topical silicone gel.

## Results

### Design

A total of 159 children with or at risk of burn scars were enrolled in the topical silicone gel and pressure garment therapy trial, of whom 52 were randomised to the topical silicone gel group, 54 to the pressure garment therapy group, and 53 to the combined topical silicone gel and pressure garment therapy group. A detailed description of the sample and clinical outcomes for each group has been reported elsewhere^7^. In summary, participants had a median (interquartile range) age of 4.9 (1.6, 10.2) years, a median (IQR) %TBSA burned of 1.0% (0.5, 3.0), and a majority were male (*n* = 99, 65%)^7^. Utility values for each group and assessment time-point are reported in Table 1 for both the primary and sensitivity (carer-perspective proxy reports) analyses. These were derived from all participants with completed assessments at baseline (n = 150, 94%), 3-months (n = 132, 83%), and 6-months (n = 119, 75%) assessments.

**Table 1:** Summary of utility values derived from the 9-item Child Health Utility (CHU-9D) instrument per group and assessment.

	Topical silicone gel	Pressure garment therapy	Combined
	Mean (95% CI)	Mean (95% CI)	Mean (95% CI)
CHU-9D utility (primary)*			
Baseline (n = 150)	0.779 (0.722, 0.831)	0.801 (0.752, 0.844)	0.817 (0.758, 0.863)
3 months** (n = 132)	0.902 (0.868, 0.936)	0.889 (0.855, 0.923)	0.904 (0.866, 0.941)
6 months** (n = 119)	0.918 (0.878, 0.959)	0.929 (0.898, 0.956)	0.926 (0.894, 0.957)
CHU-9D utility (proxy only)*			
Baseline (n = 150)	0.787 (0.762, 0.812)	0.795 (0.768, 0.819)	0.817 (0.787, 0.844)
3 months** (n = 127)	0.911 (0.879, 0.943)	0.913 (0.881, 0.945)	0.906 (0.869, 0.943)
6 months** (n = 116)	0.935 (0.898, 0.972)	0.929 (0.897, 0.961)	0.926 (0.917, 0.969)

*95% confidence intervals (CIs; bias corrected) derived from bootstrap resampling (2000 replications); **Inverse probability weighting applied to account for potential bias due to age, sex or utility (at baseline) among those retained versus lost-to-follow-up at 3 and 6 months.

### Costs

The accumulated costs for each intervention group broken down by the type of cost are presented in Table 2. Accumulated costs from the commencement of scar management to 6-months post-burn were lower in the topical silicone gel group than the pressure garment or combined groups. The mean (95% CI) total scar management cost for the topical silicone gel group was $372.87 ($337.52, $442.17) compared to $1327.02 ($1082.61, $1634.40) in the pressure garment therapy group and $1605.97 ($1075.17, $2736.11) in the combined intervention group. Participants in the groups receiving pressure garment therapy were provided with a range of 1–40 garments over the 6-months study period (median (interquartile range) = 2 (2 to 4) in both groups that received garments). Participants in the groups receiving topical silicone gel were provided with a range of zero to three 20 g tubes of topical silicone gel (median (interquartile range) = 0 (0 to 1) for both groups that received topical silicone gel) and zero to four 50 g tubes (median (interquartile range) = 0 (0 to 1) for both groups that received topical silicone gel). Splints were infrequently received by children across all groups when clinically indicated, with a range of 0–3 splints provided per participant (median (interquartile range) = 0 (0 to 0)). Costs included a soft neck splint received by one child and lower limb or upper limb thermoplastic splints received by children across all groups.

**Table 2:** Clinical costs (health service perspective) for each treatment group.

	Topical silicone gel **	Pressure garment therapy	Combined^†^
	Mean (95% CI)	Mean (95% CI)	Mean (95% CI)^⁋^
Scar management Clinic labour*	$264.69 ($233.70, 305.11)	$396.86 ($343.77, $456.35)	$546.60 ($364.12, $976.20)
Splints	$15.00 ($8.65, $22.50)	$16.63 ($10.28, $24.90)	$22.92 ($14.46, $33.75)
Pressure garments	$6.08 ($0, $22.96)	$913.53 ($701.23, $1176.69)	$966.51 ($617.16, $1646.68)
Topical silicone	$97.10 ($81.39, $113.83)	$0 ($0, $0)	$69.94 ($58.10, $84.28)
Total scar management costs	$382.87 ($337.52, $442.17)	$1327.02 ($1082.61, $1634.40)	$1605.97 ($1075.17, $2736.11)

*Includes occupational therapist labour and allied health assistants; ** Intention to treat analysis: 2 participants deviated from the topical silicone gel only (n = 1) or pressure garment only (n = 1) groups to receive combined topical silicone gel + pressure garment therapy. ^†^Combined = pressure garment therapy + topical silicone gel ^⁋^95% confidence intervals (CIs; bias corrected) derived from bootstrap resampling (2000 replications).

### Incremental cost-effectiveness

**Figure 1:**
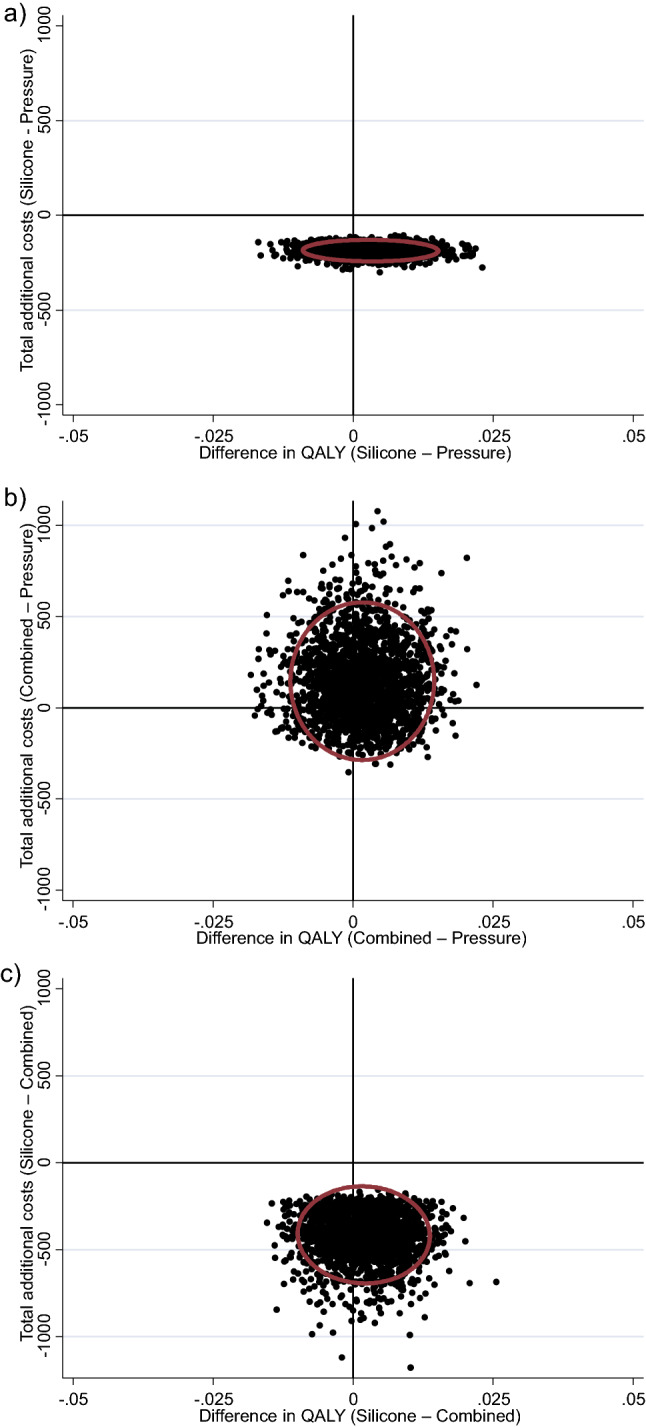
Primary analysis cost-effectiveness quadrant with 95% confidence ellipse (from bootstrap resampling) for cost (in Australian dollars) per additional Quality Adjusted Life Year (QALY) gained for: (**a**) Pressure garment therapy versus Topical silicone gel (less cost); (**b**) Combined versus Pressure garment therapy and (**c**) Combined versus Topical silicone gel (less cost).

Cost-effectiveness quadrants expressing difference in cost per difference in Quality Adjusted Life Year (QALY) gained from the primary analysis for each group comparison is presented Fig. 1. There was a greater than 99% probability that topical silicone gel was cheaper than pressure garment therapy. Mean cost savings per participant totalled $188.12 (95%CI $138.23–$252.23). There was a 70% probability that topical silicone gel therapy resulted in greater QALYs than pressure garment therapy, after adjusting for baseline QALYs. The probability that topical silicone gel therapy dominated pressure garment therapy (was cheaper and more effective) was 70%.

There was a 73% probability that pressure garment therapy was cheaper than combined therapy. Mean cost savings per participant totalled $142.89 (95% CI $-168.60–$693.94). There was a 39% probability that pressure garment therapy resulted in greater QALYs than combined therapy, after adjusting for baseline QALYs. The probability that pressure garment therapy dominated combined therapy (was cheaper and more effective) was 29%.

There was a greater than 99% probability that topical silicone gel therapy was cheaper than combined therapy. Mean cost savings per participant totalled $408.79 (95% CI $234.51–$776.26). There was a 63% probability that topical silicone gel resulted in greater QALYs than pressure garment therapy, after adjusting for baseline QALYs. The probability that topical silicone gel dominated combined therapy (was cheaper and more effective) was 63%.

### Sensitivity analysis

**Figure 2:**
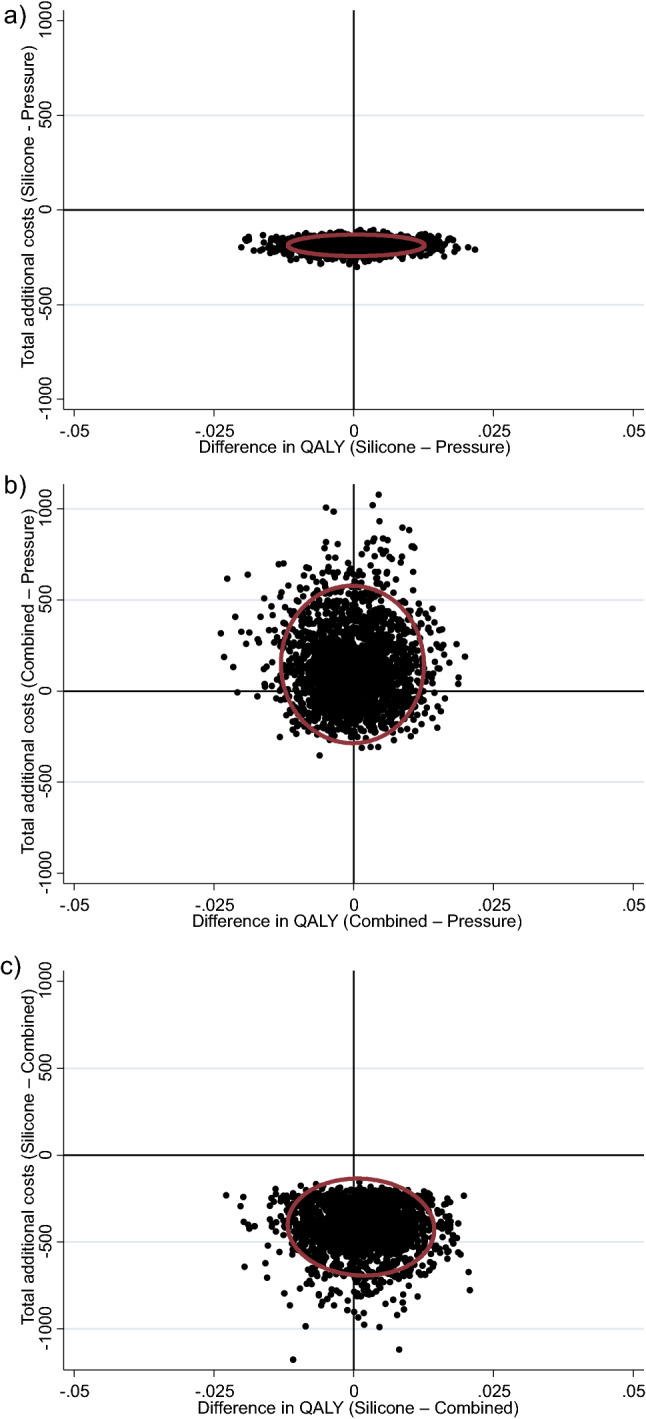
Sensitivity analysis of cost-effectiveness quadrant with 95% confidence ellipse (from bootstrap resampling) for cost (in Australian dollars) per additional Quality Adjusted Life Year (QALY) gained analysis for: (**a**) Pressure garment therapy versus Silicone (less cost); (**b**) Combined versus Pressure garment therapy and (**c**) Combined versus Silicone (less cost).

The primary analysis used child report QALY data where available and parent-proxy report where child-report data was not available. A sensitivity analysis was conducted to examine whether findings were consistent if parent-proxy reports were used for all children. Overall, findings were consistent with the primary analysis (Fig. 2). There was a greater than 99% probability that topical silicone gel was cheaper than pressure garment therapy. There was a 53% probability that topical silicone gel therapy resulted in greater QALYs than pressure garment therapy. The probability that topical silicone gel therapy dominated pressure garment therapy (was cheaper and more effective) was 53%. There was a 73% probability that pressure garment therapy was cheaper than combined therapy. There was a 52% probability that pressure garment therapy resulted in greater QALYs than combined therapy. The probability that pressure garment therapy dominated combined therapy was 37%. There was a greater than 99% probability that topical silicone gel therapy was cheaper than combined therapy. There was a 59% probability that topical silicone gel therapy resulted in greater QALYs than combined therapy. The probability that topical silicone gel dominated combined therapy was 59%.

## Discussion

Findings from this study indicated the topical silicone gel intervention was cheaper than pressure garment therapy (Figs. 1a, 2a) or the combination of topical silicone gel and pressure garment therapy (Figs. 1c, 2c). Health-related quality of life was similar across all groups at each timepoint without substantial difference in QALY estimates between groups and no clear difference between groups in QALYs in the primary analysis or the sensitivity analysis. On balance, due to patient outcomes being consistent across each group, but the topical silicone only group being cheaper than either pressure garment therapy alone or both interventions combined, findings from this study support the use of topical silicone gel as a cost-effective intervention for post-burn scar management in children. Nonetheless, in the absence of any notable difference in effectiveness, there may be other considerations beyond costs such as treatment burden that lead clinicians to tailor their choice of intervention to that deemed best for the individual patient, particularly in circumstances where cost is not the major consideration.

There have been relatively few economic evaluations investigating cost-effectiveness of paediatric burn care interventions^12,14^. This has been the first study of its kind to report the cost-effectiveness of these three scar management approaches that are commonly used as part of contemporary clinical care practices among children recovering from burns worldwide, despite pressure management having been used as first line interventions for scar management since the late 1960s^15^. These findings are likely to have important implications for cost-constrained environments where custom-made pressure garments may not be readily available, and topical silicone gel may be logistically easier to procure. However, in relation to mean total healthcare costs per burn patient of $88,218 (range $704–$717,306) reported in a 2014 systematic review, the mean scar management costs of up to $1605 (95%CI $1075–$2736) per person in the current study were relatively small, accounting for approximately 2% of total burn care^9^. Studies included in the review were similar to the current study in that they were from high income countries, mostly adopted a health care perspective, and were conducted for less than 1 year post-burn^9^.

The finding of similar health-related quality of life scores across each group in the trial was interesting to have observed. It is also noteworthy that in the trial underlying this economic evaluation, there were similar patient outcomes across a range of other scar-related clinical measures, for example, scar thickness and itch^7^. It has previously been postulated that children receiving pressure garment therapy may receive a feeling of comfort or view covering of the scar from sight favourably^16^, although in adult patients the opposite has also been reported where adults receiving pressure garment therapy may avoid seeing people or perceive other people as responding negatively to the presence of the pressure garment^17^. It is plausible that in addition to direct impact on the scar outcome, the expectations and behaviours of family members and peers, as well as the attitudes of health professionals, may influence health-related quality of life reports among people receiving interventions for scar prevention and management. However, further research is required to understand the relative differential impact of these factors on patients’ health-related quality of life in the context of different clinical interventions.

### Strengths, limitations, and future research directions

The prospective collection of costs and effects alongside a randomised trial may be considered a strength of this economic evaluation. However, it is important to recognise that this trial was focused on children with less than 40% total body surface area burns and non-facial burns. While the included sample reflects the most common types of burns presenting to hospitals, findings may not generalise to dissimilar patients who may have different or more complex scar management requirements or different acute care. The results may also not directly generalise to centres who manufacture their own pressure garments on-site, where the cost of pressure garments may be lower than custom-made pressure garments, to other topical silicone gels, or different wear and care regimes. Further, the results may not extrapolate to low- and middle-income countries where the population receiving scar management and type of interventions may differ. The high standard of acute care prior to scar management in the current study setting may have reflected the lack of differences observed between the treatment groups which may be different in low- and middle-income countries. With respect to clinical decision-making, the current cost-effectiveness evaluation may be particularly helpful for supporting use of topical silicone gel in clinical environments where engagement and self-management using pressure garment therapy is challenging.

This study examined cost-effectiveness from a health service perspective in a publicly funded healthcare system, which was appropriate for addressing the study aim informed by the clinical trial findings originating from this setting. However, other impacts on families, including work-related productivity losses of caregivers, treatment burden on caregivers of assisting the child to complete daily scar management routines and travel costs to appointments, were beyond the scope of this economic evaluation and may be a target for future research as spill-over effects onto family members have been identified in other paediatric populations^18^. The experiences of caregivers and clinicians regarding the treatment burden of pressure garment therapy has previously been reported in the PEGASUS pressure garment RCT and evaluation^19^. In the current study this burden was likely greatest in the combined pressure garment and topical silicone therapy group and least in the topical silicone gel therapy group, based on the recommended wear and care regimes and frequency of follow-up for the study interventions^7^. For example, the recommended regime for pressure therapy in two of the study groups involved the supply of two custom made pressure garments at least every 3 months (usually requiring a visit to a specialist or regional outreach centre), wearing garments for 23 h a day (including removing and reapplying clean garments once a day); and caring for the pressure garment by hand washing. In contrast the topical silicone therapy in two of the study groups involved applying a topical silicone gel one to two times daily to clean, dry, healed skin and checking the skin after 8 h of wear to determine if a second application was required^7^.

While health-related quality of life was comparable in each group within the time-horizon of this study, this does not negate the merit of examining longer time horizons in future studies of scar management interventions, and the potential for effective scar management with children to have long-lasting benefits into adulthood. This is likely to be particularly important in other intervention scenarios where additional investment in early scar prevention and management is likely to have additional long-term benefit in comparison to less resource-consuming earlier management associated with less favourable longer-term outcomes. Nonetheless, this study was successful in addressing the intended research aims and has provided new information regarding the cost-effectiveness of topical silicone gel and pressure garment therapy among children with burn-related scars.

## Methods

### Trial background

The study was registered on the Australian and New Zealand Clinical Trial Registry (ACTRN12616001100482) on 15/08/2016. Ethical approval was received from the Children’s Health Queensland Human Research Ethics Committee (HREC/15/QRCH/249), and The University of Queensland Human Research Ethics Committee (approval number 2016000558). All children aged 5 years and older provided verbal assent to participate. Written informed consent to participate in the study was obtained from a caregiver of each child. The Consolidated Health Economic Evaluation Reporting Standards (CHEERS) were followed in the study reporting^20^. All methods were performed in accordance with the relevant guidelines and regulations.

### Study design and setting

A trial-based cost-effectiveness (cost-utility) analysis was conducted from the perspective of a health service provider deciding which intervention approach to implement in a scar management clinic. Data for this economic evaluation were collected alongside a three-parallel-arm randomized controlled trial (group allocation ratio 1:1:1) conducted between August 2016 and November 2018 as recently reported^7^. The interventions were administered to children up to 18 years of age with burn injuries receiving scar interventions following spontaneous healing, skin grafting or reconstruction for a pre-existing scar with participants recruited from a metropolitan hospital, specialist paediatric burns centre in Australia. Follow-up data were collected from the burns centre, a co-located research centre, four regional hospitals located up to 620 km from the burns centre, and participant’s homes. Consistent with the primary trial end-point, the 6-months follow-up assessment post-burn injury or reconstructive surgery was adopted as the time-horizon for this economic evaluation to utilise the prospectively collected data for scar management costs and quality of life (from which QALYs were estimated). From this time in their recovery, children typically ceased accessing scar management services as their scar(s) matured.

During the study approximately 1300 children aged from birth to 16 years presenting for the first time were seen annually at the recruiting burns centre, of whom approximately one quarter accessed burn scar clinic services as part of usual care. Burn scar clinics were led by occupational therapists and supported by allied health assistants. These outpatient scar clinics are funded through the state public healthcare system and do not incur out-of-pocket costs for children or their families, including for pressure garments or topical silicone gel products used for scar management.

### Trial sample

Children were included if they: (1) were managed in the acute phase post-burn (up to 16 years of age) and received skin grafting or had spontaneously healing wounds that required ≥ 17 days to heal; (2) had an acute burn injury to 40% or less of their total body surface area burned (%TBSA); (3) were receiving reconstruction surgery for a pre-existing burn scar up to 18 years of age; (4) had a guardian who was able to provide informed consent.

Children with a cognitive impairment affecting communication were eligible to be included in the trial, although were not required to complete questionnaires. Children were excluded if they were: referred to alternative local health services prior to scar management commencing; had comorbidities potentially influencing primary trial outcomes (e.g., dermatological or neurological disorders); or had isolated facial, ear or genital burns.

### Interventions and comparators

Participants were randomised to receive one of three intervention approaches in the three arms of the randomised trial, respectively: (1) topical silicone gel only (medical use, Strataderm^®^, Stratpharma, Basel, Switzerland); (2) pressure garment only (Therapeutic Support Laboratory^®^ (TSL), Abbortsford, Victoria, Australia); or (3) combined topical silicone gel (Strataderm^®^) and pressure garment therapy (Therapeutic Support Laboratory, TSL^®^). These interventions were components of usual care at the participating burns centre and are consistent with intervention approaches used by most burn centres in developed countries^21,22^. Detailed information regarding these interventions has been published previously^7^. Custom-made pressure garments were manufactured off-site by TSL^®^. Topical silicone gel was supplied in either 20 g and 50 g tubes and dispensed by the occupational therapists in the participating clinic who provided advice on topical application consistent with the manufacturers guidelines.

### Intervention effect outcome

The primary measure of effect was estimates of between group differences in QALYs accrued between baseline assessment conducted in the scar management clinic (4 weeks post-burn or reconstructive surgery) and the study time-horizon (6-months follow-up). QALYs are widely used for effect estimates in economic evaluations and are a measure of disease burden that include quality of life experienced over an observed time-period, whereby one QALY represents the equivalent of 1 year lived in perfect health. QALYs are most-commonly estimated from multi-attribute utility scores (anchored by the health states of perfect health (1.00) and death (0.00)) derived from the application of preference weights to longitudinal health-related quality of life assessments. The 9-item Child Health Utility (CHU-9D) is a generic measure of health-related quality of life that can be used for comparisons across conditions, settings and management; and can be used to compare healthy and unwell children^23^. The CHU-9D is suitable for use in economic evaluations as it permits QALYs to be estimated from longitudinal assessments in clinical trials^24^ for use in cost-utility analyses through the application of societal preference weights^25,26^.

Estimating QALYs from clinical trial assessments among children is methodologically challenging. Children recovering from burns who are willing and able to self-report their health state using the CHU-9D^27,28^ are likely to have valuable insight based on their lived experiences, but not all children are able to self-report (e.g., infants, very young children, or children with cognitive impairment). On the other hand, each child participant is likely to have a parent or carer who is able to provide valuable insight from their own perspectives of the child’s lived experiences through completing the CHU-9D as a proxy-reporter. For the primary estimates of effect in this economic evaluation, child self-report of the CHU-9D (baseline assessment, 3-months, 6-months) was used whenever a child self-report was available, and proxy-report (parent / carer perspective) was used when no child report was available. In addition, sensitivity analyses were conducted to examine whether findings were robust to use of the parent / carer perspective (only), which were also recorded for all children at each assessment. This proxy-only sensitivity analyses enabled us to examine whether findings were consistent when a parent/carer proxy-only perspective was adopted, as the parent/carer viewpoint is likely to be influential during shared decision-making regarding selection of scar management approaches for children.

### Resource use and costs

Healthcare resource use related to scar management was recorded from the baseline assessment to the 6-months follow-up for each participant from the perspective of the health service provider and costed in 2018 Australian dollars. Resource use and costs were recorded during the trial data collection by occupational therapists providing clinical care and research staff with access to medical records and health service clinical costing data. This included trial intervention costs for each group (e.g., the number and volume of topical silicone gel and pressure garment therapy products), other burn scar rehabilitation related resource use and costs (e.g., for splinting) and burn scar clinic labour costs attributable to each participant (e.g., time recorded in minutes for occupational therapists and allied health assistants attributable to each participant). Labour time attributed to each participant comprised time taken to schedule appointments, provide trial intervention(s) including measuring and fitting pressure garments, education regarding wear and care of pressure garments or application of topical silicone gel or moisturisers, time taken to fabricate splints (if relevant), as well as any other within-clinic clinical care-related activity including time to record information in patients’ medical records. Labour resource use was costed using 2018 industrial award rates for occupational therapists and allied health assistants (respectively) working in public health facilities in the state where the study was conducted. Hospital-based costing data was used for thermoplastic splint cost estimates at $45 per splint and soft collar neck splints $5 per soft collar. No discounting was applied, as the time-horizon was less than 1 year.

### Analytical methods

Conventional descriptive statistics were used to describe the sample and healthcare resource use (and costs) for each trial arm. To provide unbiased estimates of effects in the presence of any missing (follow-up) utility scores at 3- and 6-months assessments, inverse probability weights^29,30^ were applied both to mean estimates per group at each time-point, as well as between group comparisons (described below) for the primary and sensitivity analyses. The use of inverse probability weights for missingness requires a two-step process^29–31^. First, a logistic regression (age, sex and baseline health utility as fixed effects) was used to predict which participants were more likely to be missing health utility estimates at each follow-up assessment^29,30^. From these logistic regressions, the probability of each participant missing utility data at each of the follow-up assessments was then calculated and inverted^29,30^ to create a weight that was applied during analyses that included 3- and 6-months assessments. Resource use and cost data were available for all included participants.

Paired comparisons of costs and QALYs were made between each of the trial arms and plotted on cost-effectiveness quadrants for the primary and sensitivity analyses. Due to the potential for small, but potentially influential between group differences in health utility at baseline, between group differences in QALYs were estimated with the use of regression analyses that adjusted for baseline utility assessments consistent with prior recommendations^24^. The difference in cost and QALYs for each paired comparison was estimated from the respective regression coefficients. Bootstrap resampling (2000 replications of the sample stratified by group) was used to quantify estimates of uncertainty (confidence intervals for between group differences in costs and QALYs, and confidence ellipses for cost-effectiveness estimates). This included estimating the probability that each intervention was cheaper and yielded more QALYs than the comparator for each of the between-group comparisons. Cost and effectiveness estimates (with 95% confidence ellipses) were visualised on a cost-effectiveness plane for each between-group comparison, whereby the lower two quadrants represented cost savings, with right lower quadrant representing dominance (i.e., cheaper and more effective). It was intended that incremental cost effectiveness ratios (ICERs) would be used to express cost-per-QALY gained if differences in QALY and cost estimates were observed between groups using the formulae:

- ICER (silicone vs pressure) = Cost difference (silicone—pressure)/QALY difference (silicone—pressure)
- ICER (silicone vs combined) = Cost difference (silicone—combined)/QALY difference (silicone—combined)
- ICER (pressure vs combined) = Cost difference (pressure—combined)/QALY difference (pressure—combined)

All analyses were conducted for the primary QALY estimates, as well as the proxy-only sensitivity analyses. Statistical analyses were conducted using Stata 13 (StataCorp, College Station, Texas).

## Data Availability

Data are available upon request from the lead author Professor Steven McPhail if the appropriate permissions are obtained including approval by Children’s Health Queensland Human Research Ethics Committee and if the conditions for access are agreed to.
